# Risks and Benefits of Weight Gain in Children With Undernutrition

**DOI:** 10.1001/jamanetworkopen.2025.14289

**Published:** 2025-06-06

**Authors:** Beverly I. Strassmann, Claudius Vincenz, Eduardo Villamor, Jennie L. Lovett, Zachary D. Dolo, Kerby Shedden

**Affiliations:** 1 Department of Anthropology, University of Michigan, Ann Arbor; 2 Research Center for Group Dynamics, Institute for Social Research, University of Michigan, Ann Arbor; 3 Department of Epidemiology, University of Michigan School of Public Health, Ann Arbor; 4 Frankel Institute for Heart and Brain Health, University of Michigan Medical School, Ann Arbor; 5 Dogon Longitudinal Study, Bandiagara Region, Republic of Mali; 6 Department of Statistics, University of Michigan, Ann Arbor

## Abstract

**Importance:**

Previous studies in low-resource settings have emphasized the risks of childhood weight gain for increased body mass index (BMI) and systolic blood pressure (SBP) in adulthood. However, these studies have not directly compared the risk of extra weight against the benefit of increased adult height.

**Objective:**

To test the hypothesis that a continuous 1-SD increase in weight from age 1 to 10 years was associated with taller stature in adulthood but not with increased risk for obesity or hypertension.

**Design, Setting, and Participants:**

This prospective cohort study, called the Dogon Longitudinal Study, is a 21-year population-based multigenerational cohort study conducted from 1998 to 2019 in 9 Dogon villages on the Bandiagara Escarpment in Mali. A mediation analysis was conducted in 2024 to calculate the estimated total effect on adult SBP of a 1-SD weight increase over the mean throughout the first decade of childhood. This estimated total effect was decomposed into estimated direct and indirect effects. Children aged 5 years or younger on April 15, 1998, as well as all children born from that date to July 2, 2000, were eligible to participate in the F1 generation.

**Exposures:**

Weight and height trajectories from age 1 to 10 years.

**Main Outcomes and Measures:**

For the mediation analysis, adult SBP was the outcome, and the mediators were height and BMI at age 21 years. The mediation analysis used linear mixed models for SBP, adult height, and adult BMI.

**Results:**

A total of 1348 participants (645 females [47.8%], 703 males [52.2%]) of the F1 generation contributed 10 081 SBP measurements to the analyses. These participants completed the study and had a median (IQR) of 12 (11-14) follow-up visits from enrollment (at median [IQR] age of 1.59 [0.62-3.44] years) to last measurement (at median [IQR] age of 21.14 [19.47-23.14] years). After adjusting for both parents’ height and SBP (F0 generation), analyses included 433 females and 501 males, with 3384 and 3770 SBP measurements, respectively. The total effect on adult SBP of being 1 SD above the mean, instead of at the mean, childhood weight trajectory was 1.9 (95% CI, 0.9-2.8) mm Hg for females and 3.2 (95% CI, 2.3-4.2) mm Hg for males. This total effect was mediated by an indirect effect through adult height of 2.3 (95% CI, 0.9-3.7) mm Hg in females and 3.9 (95% CI, 2.4-5.4) mm Hg in males and by an indirect effect through adult BMI of 2.6 (95% CI, 2.0-3.2) mm Hg in females and 1.4 (95% CI, 0.6-2.2) mm Hg in males. The direct effect on SBP was −3.1 (95% CI, −4.5 to −1.6) mm Hg in females and −2.1 (95% CI, −3.2 to −0.9) mm Hg in males. A 1-SD weight increase in childhood was associated with a 1.6% increase in the prevalence of obesity in females and no increase in the prevalence of obesity in males. The percentage of individuals whose SBP was 130 mm Hg or higher increased by 0.5% in females and 3.7% in males. The mean (SE) height at age 21 years increased by 3.0 (0.5) cm in females and 4.1 (0.6) cm in males.

**Conclusions and Relevance:**

The findings of this cohort study of an undernourished population in Mali supported the hypothesis, suggesting that the risks of 1 SD in childhood weight gain for hypertension and obesity in adulthood were small compared with the benefits of taller stature.

## Key Points

**Question:**

Where stunting is prevalent, is a 1-SD increase in childhood weight associated with adults with taller stature but not with increased risk for obesity and hypertension?

**Findings:**

In this cohort study of 1348 participants, mediation analysis showed that a 1-SD weight increase from age 1 to 10 years was associated with gains in adult stature for females and males by age 21 years. Increased risk for obesity and hypertension was negligible.

**Meaning:**

In settings with an undernourished population, sound public health policy should balance the benefits of childhood weight gain for increased adult stature against the risks of obesity and hypertension.

## Teaser

This cohort study examines the association of weight and height trajectories during the first decade of life with risk for obesity or hypertension by 21 years of age among individuals residing in a region of Mali where undernourishment is common.

## Introduction

Globally, elevated blood pressure is the single greatest modifiable risk factor for disability and deaths from cardiovascular disease.^1^ The tracking of childhood body size into adult body size is one aspect of the cardiovascular disease epidemic.^2,3,4^ Tracking is particularly problematic in obesogenic environments where children with increased weight are at risk for higher body mass index (BMI) and elevated systolic blood pressure (SBP) in adulthood.^5^ In low- and middle-income countries, concerns about the tracking of body size have led to public health advice against rapid relative weight gain in children after age 2 years.^6^ However, children’s linear growth deficit can accumulate beyond the first 2 years of life,^7^ and persistent undernutrition has many disadvantages. The costs include immunodeficiency,^8^ lower cognitive function,^9,10,11,12,13^ not reaching adult height potential,^14^ reduced wages,^15,16,17^ increased risk for metabolic disease,^8^ and higher morbidity and mortality.^8,18^

Interventions to prevent undernutrition in the first 1000 days of life are beneficial for health and human capital,^6^ but many children are not reached during this early period. Research is therefore needed on the potential to improve growth beyond age 2 years.^7,19^ In undernourished populations, the risks of childhood weight gain for increased BMI and SBP in adulthood must be compared with the benefits of childhood weight gain for growth in stature. To inform public health policy for childhood undernourishment, we used mediation analysis to test the hypothesis that a continuous 1-SD increase in weight from age 1 to 10 years (as measured by internal variation) was associated with taller stature in adulthood but not with increased risk for obesity or hypertension.

This study uses primary data from the Dogon Longitudinal Study, which is a multigenerational cohort study that we conducted in Mali, West Africa, from 1998 to 2019. This study has a unique dataset in that we followed up children (F1 generation) prospectively for 21 years, from a median (IQR) age of 1.59 (0.62-3.44) years to 21.14 (19.47-23.14) years, during which time we collected repeated measures of body size and blood pressure. Compared with the World Health Organization reference population,^20^ in the Dogon population, 58% of the girls and 59% of the boys were stunted at age 2 years, with 22% of the girls and 29% of the boys being severely stunted. Using these longitudinal data, we provide new evidence to inform policy directed at benefiting the lives of the 149.2 million children under 5 years of age with stunting.^14^

## Methods

### Study Design and Population

The 9 villages included in the Dogon Longitudinal Study belonged to a single Dogon community on the Bandiagara Escarpment in Mali. Children aged 5 years or younger on April 15, 1998, and all children born from that date to July 2, 2000, were eligible to participate in the F1 generation. In this population-based study, all parents of eligible children agreed to enroll their children. We measured body size and assessed covariates in 1998, 1999, 2000, 2004, and 2007 and annually from 2010 to 2019. We measured each participant’s weight (kg) and height (cm) on approximately 12 occasions and SBP on approximately 9 occasions. Prior to 2010, follow-up of the cohort took place in the original study villages; from 2010 onward, annual follow-up took place in the villages and in Bamako, the primary destination for participants who migrated. All data were collected with oral or written informed individual consent or assent. When oral consent was used, it was justified by limited literacy, cultural appropriateness, and minimal risk. The Dogon Longitudinal Study was approved by the University of Michigan Institutional Review Board (IRB) and the Malian IRB in conjunction with annual approval by the Malian government from 1998 to 2019. Annual ethical approval was also obtained at the community level in Mali. We followed the Strengthening the Reporting of Observational Studies in Epidemiology (STROBE) reporting guideline for cohort studies.^21^

### SBP and Body Measurements 

We measured SBP (mm Hg) in triplicate on the right arm after a 20-minute seated rest. The auscultation method and manual blood pressure monitors (Omron Healthcare) with appropriate cuff sizes were used according to international recommendations.^22^

Body size was measured on enrollment and in each round of follow-up. Participants were weighed to the nearest 0.1 kg using battery-powered electronic scales (Tanita Corp; Seca). Height was measured in triplicate to the nearest 0.1 cm using a portable stadiometer (Perspective Enterprises), and the 3 values were averaged. Body mass index was calculated as weight in kilograms divided by height in meters squared.

### Covariates

Covariates assessed at the time of enrollment included the village of residence and the *z* score for the wealth rank of each participant’s family relative to other families in the same village (eMethods in Supplement 1).^23^ Time-varying covariates assessed at each measurement session included ambient temperature (Celsius [°C]) and the number of cigarettes smoked per week, which was consistently 0 for females. To reduce error from white-coat hypertension, our models adjusted for the log of the cumulative number of occasions on which the study team had measured each person’s blood pressure. Instead of deleting pregnant women’s data, which would have reduced the representativeness of the findings in this high-fertility population, we adjusted for the number of months of pregnancy, which we back calculated from dates of birth for F2 offspring. Similarly, we adjusted for the number of months of breastfeeding at the time of each measurement session, which we estimated from interview data and dates of offspring birth.

### Statistical Analysis

**Figure 1.**
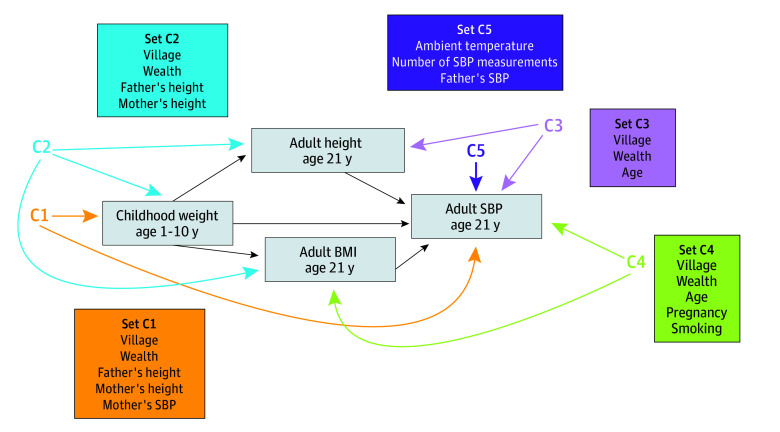
Childhood Weight and Adult Systolic Blood Pressure (SBP) Mediated by Adult Height and Adult Body Mass Index (BMI) Measured potential common causes of childhood weight and adult SBP (Set C1), childhood weight, adult BMI, and adult height (Set C2), adult SBP and adult height (Set C3), adult SBP and adult BMI (Set C4), and extraneous variables (Set C5). Childhood height as the exposure has the same causal association structure.

We conducted a mediation analysis to quantify the direct and indirect associations between childhood weight (the exposure) and adult SBP at age 21 years (the outcome), mediated by adult height and adult BMI at age 21 years (Figure 1). The exposure variable was the trajectory of childhood weight from 1 to 10 years of age. We modeled the effect of changing the childhood growth trajectory from the mean trajectory to 1 SD above the mean trajectory, continuously from age 1 to 10 years. At each age, we modeled a 1-SD increase in childhood weight that was sex specific and internal to the data. In a secondary analysis, we substituted childhood height for childhood weight.

To obtain body size (weight or height) measures at identical ages for analysis, we multiply imputed childhood body measurements at integer ages 1 through 10 years based on all directly observed measurements between ages 0 and 11 years. Imputation was based on a continuous time gaussian process model^24^ (eMethods and eFigure 1 in Supplement 1). Since annual body size measures were serially associated, we calculated their principal components (PCs) and summarized the trajectories of childhood growth using the PC scores.

The mediation analysis followed a model-based approach,^25^ using linear mixed models for SBP, adult height, and adult BMI. The model was based on annual measurements of these 3 variables from a median age of 14 to 21 years (Table). Adult blood pressure was regressed on the PC scores for the exposure (weight or height in childhood) in linear mixed models. We considered models that included 1, 2, or 3 PCs and selected the best fitting number of PCs to include using the Akaike Information Criterion.^26^ The mediation analyses included the measured potential common causes of the exposure and the outcome, the mediators and the outcome, the exposure and the mediators, and extraneous variables (Figure 1).

**Table.** Characteristics of F1 Generation Participants and Their F0 Generation Parents at the 50th (10th-90th) Percentiles

Characteristic	F1 longitudinal data				F0 cross-sectional data	
	First SBP measurement		Last SBP measurement		Mothers (n = 552)	Fathers (n = 418)
	Females (n = 433)	Males (n = 501)	Females (n = 433)	Males (n = 501)		
Age, y	14 (11-17)	14 (11-17)	21 (18-24)	21 (19-24)	44 (35-55)	50 (40-65)
SBP, mm Hg	107 (95-119)	102 (91-116)	104 (93-119)	108 (97-123)	108 (95-129)	120 (103-148)
Height, cm	144.6 (130.1-158.3)	141.7 (130.3-160.3)	158.5 (150.7-165.9)	169.0 (160.1-176.7)	158.5 (152.1-165.3)	168.4 (160.7-176.3)
BMI	16.4 (14.1-20.8)	16.0 (14.4-18.6)	22.0 (19.0-25.6)	20.8 (18.2-23.5)	21.3 (18.5-25.0)	21.8 (18.5-25.5)

Abbreviations: BMI, body mass index (calculated as weight in kilograms divided by height in meters squared); SBP, systolic blood pressure.

To avoid collider-stratification bias^27,28^ in the estimation of total effects, we avoided adjustment for covariates that could be affected by the participant’s body size trajectory in the first decade of childhood. In a sensitivity analysis, we created models that minimally adjusted for covariates. We included nonlinear age terms as well as exposure (childhood weight or height) by mediator (adult BMI and height) interactions. Independent random effects for participant and mother along with a random slope for age within participants accounted for dependencies in the blood pressure measurements within participants and between siblings.

The clinical significance of a 1-SD weight increase over the mean during the first decade of childhood was assessed by estimating the proportion of people whose adult SBP or BMI exceeded thresholds for hypertension, overweight, and obesity. The analyses were conducted from January to August 2024 using Python, version 3.11, and statsmodels, version 0.14.3 (Python Software Foundation). Statistical significance was set at *P* < .05, and all tests were 2-tailed.

## Results

### Participants

**Figure 2.**
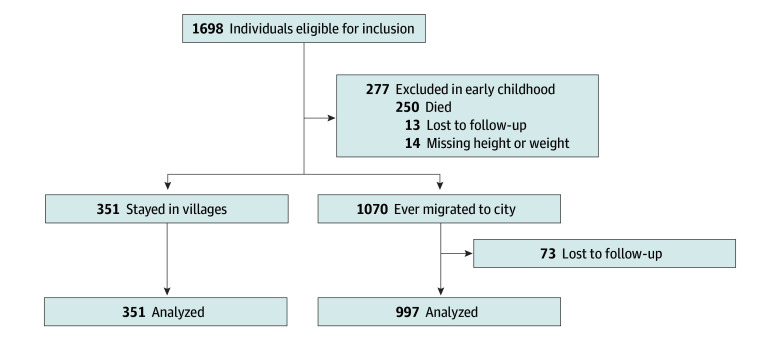
Study Flow Diagram Sample sizes for retention and attrition in the villages and the city are shown. Sample sizes for retention and attrition in the villages and the city, producing a total of 1348 participants in the study.

A total of 1698 individuals met the eligibility criteria and were included in the F1 cohort. There was attrition in the study villages (n = 277) and after urban migration (n = 73) (Figure 2). Bamako was the primary destination for the 1070 participants who migrated to a city, either temporarily or permanently; 997 (93.2%) of these migrants were retained in the study. Combining individuals in the villages with those in Bamako yielded a total of 1348 participants (79.4% of the original cohort), who contributed 10 081 SBP measurements. These 1348 participants included 645 females (47.8%) and 703 males (52.2%) with a median (IQR) of 12 (11-14) follow-up visits from study enrollment (at median [IQR] age of 1.59 [0.62-3.44] years) to last measurement (at median [IQR] age of 21.14 [19.47-23.14] years). The 25th, 50th, and 75th percentile for follow-up time was 18.5, 19.9, and 20.5 years for females and 18.4, 19.7, and 20.6 years for males, respectively. We measured F0 parents cross-sectionally using the same protocols as for the F1 participants. A total of 433 females (with 3384 SBP measurements) and 501 males (with 3770 SBP measurements) of the F1 generation had data for both parents and were included in the main analyses (Table).

The Table shows the characteristics of participants on first and last SBP measurement as well as the anthropometric and SBP data for their parents. On last SBP measurement, which took place at a median (IQR) age of 21.26 (19.51-23.17) years for females and 21.01 (19.44-23.05) years for males, the 10th, 50th, and 90th quantiles for SBP were 93, 104, and 119 mm Hg for females and 97, 108, and 123 mm Hg for males, respectively. The 10th, 50th, and 90th quantiles for BMI were 19.0, 22.0, and 25.6 for females and 18.2, 20.8, and 23.5 for males, respectively. Additionally, on last measurement, the median (10th-90th percentile) height for F1 females was 158.5 (157.0-165.9) cm and for their F0 mothers was also 158.5 (152.1-165.3) cm, and the median height for F1 males was 169.0 (160.1-176.7) cm, which was 0.6 cm taller than for their F0 fathers, whose height was 168.4 (160.7-176.3) cm (Table). The observed prevalence of prehypertension and hypertension is shown in eResults in Supplement 1.

### Mediation Analysis

**Figure 3.**
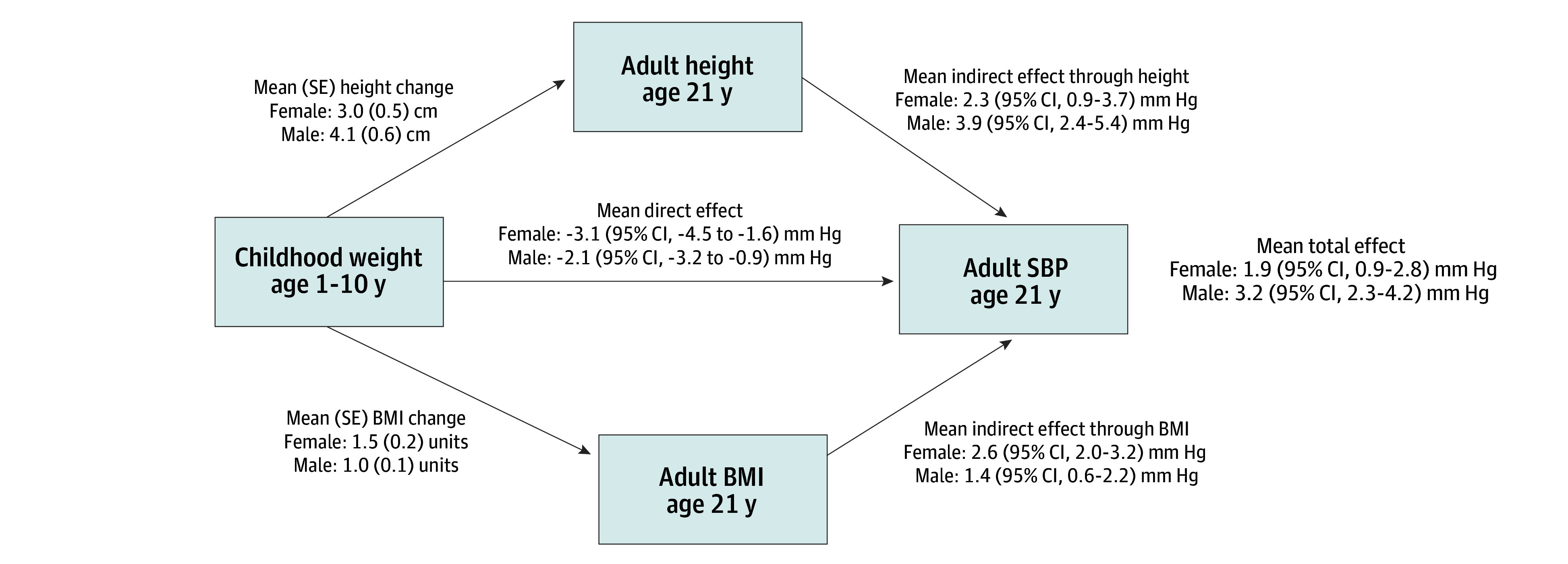
Mediation Analysis With Childhood Weight as the Exposure, Adult Systolic Blood Pressure (SBP) as the Outcome, and Adult Height and Adult Body Mass Index (BMI) as Mediators The exposure was modeled as the mean vs 1 SD above the mean continuously from age 1 to 10 years, and it was age- and sex-specific as well as internal to the data. The direct and indirect effects were estimated at age 21 years. The model was adjusted for the exposure-outcome confounders, exposure-mediator confounders, mediator-outcome confounders, and extraneous variables shown in Figure 1 and eMethods and eTables 1 to 3 in Supplement 1.

Figure 3 shows the results of a mediation analysis with 1-SD over-the-mean increase in childhood weight as the exposure, controlling for the confounders and extraneous variables shown in Figure 1. Based on Akaike Information Criterion, only 1 PC was included in all models; PC loadings are provided in eFigure 2 in Supplement 1. Full model specification is in eMethods and eTables 1 to 3 in Supplement 1.

The model estimated that females who were 1 SD above the mean weight instead of being at the mean weight in childhood (at ages 1 to 10 years) had higher SBP (estimated total effect at age 21 years) by a mean of 1.9 (95% CI, 0.9-2.8) mm Hg (Figure 3). We decomposed this estimated total effect of childhood growth on adult SBP into 2 indirect effects (1 through adult height, and 1 through adult BMI) and a direct effect. The indirect effect through adult height consisted of a mean (SE) increase of 3.0 (0.5) cm in height associated with a mean increase of 2.3 (95% CI, 0.9-3.7) mm Hg in adult SBP. The indirect effect through adult BMI consisted of a mean (SE) increase of 1.5 (0.2) units in adult BMI associated with a mean increase of 2.6 (95% CI, 2.0-3.2) mm Hg in adult SBP. The direct effect was a mean of −3.1 (95% CI, −4.5 to −1.6) mm Hg in adult SBP. The negative direct effect exceeded in magnitude the mean increase in SBP mediated by increased BMI, although not the increase in SBP mediated by BMI and height combined (Figure 3).

Males who were 1 SD above the mean weight instead of being at the mean weight in childhood (at ages 1 to 10 years) were estimated to have higher adult SBP by a mean of 3.2 (95% CI, 2.3-4.2) mm Hg (total effect). The indirect effect through adult height consisted of a mean (SE) increase of 4.1 (0.6) cm in height associated with a mean increase of 3.9 (95% CI, 2.4-5.4) mm Hg in adult SBP. The indirect effect through adult BMI consisted of a mean (SE) increase of 1.0 (0.1) units in adult BMI associated with a mean increase of 1.4 (95% CI, 0.6-2.2) mm Hg in adult SBP. The direct effect was a mean of −2.1 (95% CI, −3.2 to −0.9) mm Hg in adult SBP. Similar to females, in males this negative direct effect more than offset the increase in SBP mediated by increased BMI, although not the increase in SBP mediated by the combination of BMI and height (Figure 3).

**Figure 4.**
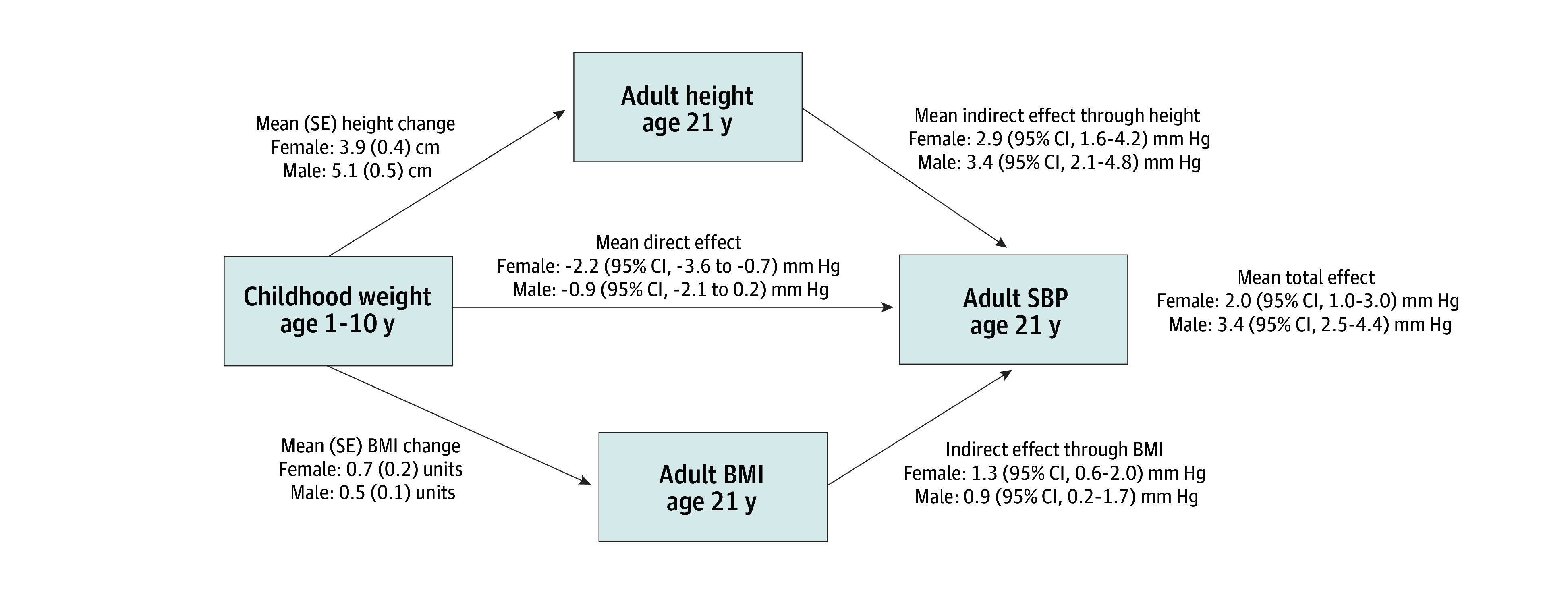
Mediation Analysis With Childhood Height as the Exposure, Adult Systolic Blood Pressure (SBP) as the Outcome, and Adult Height and Adult Body Mass Index (BMI) as Mediators The exposure was modeled as the mean vs 1 SD above the mean continuously from age 1 to 10 years, and it was age- and sex-specific as well as internal to the data. The outcome and mediators were for adults at age 21 years. The model was adjusted for the exposure-outcome confounders, exposure-mediator confounders, mediator-outcome confounders, and extraneous variables shown in Figure 1 and eMethods and eTables 4 to 6 in Supplement 1.

When childhood height (1 SD above the mean) was used as the exposure instead of childhood weight, adult height increased by a mean (SE) of 3.9 (0.4) cm in females and 5.1 (0.5) cm in males (Figure 4; eTables 4-6 in Supplement 1). In a sensitivity analysis for childhood weight and height that minimally adjusted for covariates, the results were similar to the main models except that the increase in adult stature was greater and the increase in adult BMI and SBP was smaller than in the fully adjusted models (eFigures 3 and 4 in Supplement 1).

In the observed sample at age 21 years, we estimated the prevalence of SBP of 130 mm Hg or higher to be 3.0% for females and 4.1% for males. On the 1-SD increase in childhood weight trajectory, these values became 3.5% in females and 7.8% in males. Moreover, in the observed population, the estimated prevalence of BMI of 25 or higher was estimated to be 15.6% for females and 1.6% for males; the prevalence of BMI of 30 or higher was estimated to be 0% for both sexes. On the 1-SD above-the-mean trajectory, these values in females and males became 31.2% and 3.1%, respectively, with the prevalence of obesity at 1.6% for females and 0% for males. Thus, the main implication for females was that an additional 15.6% crossed the cutoff for BMI classification as overweight. The main implication for males was that an additional 3.7% attained an SBP of 130 mm Hg or higher.

## Discussion

In this prospective cohort study, the Dogon Longitudinal Study, we collected longitudinal primary data from participants whom we followed up regularly from a median age of 1.59 to 21.14 years. We used mediation analysis to test the estimated effects of a hypothetical intervention in which the weight distribution for the children was increased by a constant increase of 1 SD over the mean at each age from 1 to 10 years. Our hypothesis was that the intervention permitted children to reap the benefit of becoming taller as adults, with relatively few of them becoming overweight, obese, or hypertensive. The results supported this hypothesis for both females and males.

### Females in the Mediation Model 

In the mediation model, females who were on the childhood weight trajectory that was 1 SD above the mean had higher total SBP at age 21 years by a mean of 1.9 (95% CI, 0.9-2.8) mm Hg. This increase in SBP was estimated to put an additional 0.5% of females at or above the threshold of SBP of 130 mm Hg or higher at age 21 years, which is the middle of the range for prehypertension (120-139 mm Hg).^29^ The risk of this increase of half a percent is minimal or even negligible compared with the advantages of increased height, which was expected to increase by a mean (SE) of 3.0 (0.5) cm across females.

Taller maternal stature has been associated with reduced risk of maternal and neonatal death due to pelvic obstruction in childbirth.^18^ In low-income countries, pelvic obstruction is a leading cause of maternal mortality, and taller mothers are likely to have healthier offspring.^18^ Analysis of 109 demographic and health surveys in 54 countries showed that even a 1-cm increase in maternal stature was inversely associated with offspring mortality, underweight, and stunting in infancy and childhood.^30^

Females who were on the childhood weight trajectory that was 1 SD above the mean were estimated to have a mean (SE) BMI that was 1.5 (0.2) units higher at age 21 years. This increase in BMI was estimated to increase the prevalence of overweight (BMI ≥25) in females by 15.6 percentage points. Meanwhile, the estimated increase in the prevalence of obesity was only 1.6 percentage points.

### Males in the Mediation Model 

In our mediation model, males who were on the childhood weight trajectory that was increased by 1 SD above the mean were estimated to have higher SBP at age 21 years by a mean of 3.2 (95% CI, 2.3-4.2) mm Hg. Their mean (SE) height was estimated to increase by 4.1 (0.6) cm and their mean (SE) BMI by 1.0 (0.1) units. The increase in SBP was estimated to put an additional 3.7% of males at or above the threshold of SBP of 130 mm Hg or higher at age 21 years. However, most of the estimated increase in SBP in males on the higher childhood weight trajectory was due to their increased height rather than an increase in BMI. In addition to the health benefits of better early linear growth,^8^ taller persons tend to reap many advantages throughout their lives. One of these advantages is the height premium, or the favorable association between height and wages, which is usually reported more in men than women.^31^ Taller persons also tend to have higher educational attainment.^15,32^

Mechanistically, a negative direct association between childhood weight and adult SBP could be attributed to physiological changes that occurred during childhood or to characteristics that were carried forward from the prenatal period. For example, larger body size in childhood might be inversely associated with severe diarrhea and dehydration, which damage the kidneys and increase SBP. Better nutrition in utero is associated with higher nephron numbers in offspring and lower risk for kidney disease and hypertension in adulthood.^33^

### Previous Studies

To our knowledge, this study is the first to disentangle direct associations between childhood body size and adult SBP as well as indirect associations through both adult height and adult BMI in a human population. This approach is especially critical in populations in which undernutrition is arguably as problematic for health as high blood pressure.

Related studies that used mediation analysis did not consider height and focused only on BMI as a mediator. For example, the Generation XXI Birth Cohort Study in Portugal used mediation analysis to untangle direct and BMI-mediated associations of birth weight with childhood SBP and other cardiometabolic indicators.^3^ The Avon Longitudinal Study of Parents and Children investigated the relationship between maternal prepregnancy BMI and the mediating effect of childhood and adolescent BMI on offspring blood pressure at age 18 years.^34^ Similarly, in 3 Brazilian birth cohorts, mediation analysis was used to show that the total effect of maternal prepregnancy BMI on offspring blood pressure and non–high-density lipoprotein cholesterol was mediated by offspring BMI.^35^ Previous studies of the developmental origins of SBP in Africa also focused on the pathway to high blood pressure through childhood BMI or weight gain.^36,37,38^ Our study contributes to this literature by considering adult height and BMI simultaneously as mediators of the association between childhood body size and SBP. In so doing, we found support for our hypothesis that a 1-SD increase in childhood weight made children taller as adults with relatively few becoming overweight, obese, or hypertensive.

### Limitations

An assumption of mediation analysis is that there are no unmeasured confounders between the exposure, mediators, and the outcome.^39^ An unmeasured confounder that was a concern in the Avon Longitudinal Study of Parents and Children was the shared genetic risk of obesity and elevated blood pressure between mother and offspring.^34^ However, we were able to adjust for the height and SBP of both parents, reducing the potential for confounding by genetics. We also adjusted for numerous time-varying covariates seldom available in longitudinal studies. A sensitivity analysis showed that the results were similar in more fully and less fully adjusted models. Nonetheless, since residual confounding is always a possibility, a nutritional intervention to increase childhood body size might not have the same implications for adult outcomes as found in this study. In the Dogon population we studied, approximately 59% of the children were stunted at age 2 years. The results of this mediation analysis pertain to that context and should not be construed as generalizable to populations that are better nourished.

## Conclusions

In this cohort study of the Dogon of Mali, children with a 1-SD weight increase over the mean in the first decade of childhood were taller and had higher BMI and SBP by age 21 years. However, of these children, none became obese and few became hypertensive in adulthood. Stopping interventions to alleviate childhood undernutrition after age 2 years could be associated with shorter stature in adults whose lower SBP is substantially associated with shorter stature rather than lower BMI alone. For children with stunting and who missed the opportunity for better nourishment in utero, sound public health policy should balance the benefits of weight gain to increase adult stature against the risks of obesity and hypertension.

## Notes

Supplement 1.
eMethods

eResults
**eFigure 1.** Selected Goodness of Fit Plots for Multiple Imputation **eFigure 2.** Principal Component (PC) Loadings for Weight and Height, Obtained Separately for Females and Males, Plotted as Functions of Age **eFigure 3.** Minimally Adjusted Mediation Analysis Results With Childhood Weight as the Exposure, Adult SBP as the Outcome, and Adult Height and Adult BMI as Mediators **eFigure 4.** Minimally Adjusted Mediation Analysis Results With Childhood Height as the Exposure, Adult SBP as the Outcome, and Adult Height and Adult BMI as Mediators **eTable 1.** Predictors of Systolic Blood Pressure in the Childhood Weight Model (Figure 3 in main text) **eTable 2.** Predictors of Height in the Childhood Weight Model (Figure 3 in Main Text) **eTable 3.** Predictors of Body Mass Index in the Childhood Weight Model (Figure 3 in Main Text) **eTable 4.** Predictors of Systolic Blood Pressure in the Childhood Height Model (Figure 4 in Main Text) **eTable 5.** Predictors of Height in the Childhood Height Model (Figure 4 in Main Text) **eTable 6.** Predictors of Body Mass Index in the Childhood Height Model (Figure 4 in Main Text)

Supplement 2.
Data Sharing Statement
