# Supplementary material for: Risks and Benefits of Weight Gain in Children With Undernutrition
Source: JAMA Netw Open. 2025 Jun 6;8(6):e2514289. doi: 10.1001/jamanetworkopen.2025.14289 (PMC12144626; doi:10.1001/jamanetworkopen.2025.14289)
Supplement: Supplement 1. — eMethods eResults eFigure 1. Selected Goodness of Fit Plots for Multiple Imputation eFigure 2. Principal Component (PC) Loadings for Weight and Height, Obtained Separately for Females and Males, Plotted as Functions of Age eFigure 3. Minimally Adjusted Mediation Analysis Results With Childhood Weight as the Exposure, Adult SBP as the Outcome, and Adult Height and Adult BMI as Mediators eFigure 4. Minimally Adjusted Mediation Analysis Results With Childhood Height as the Exposure, Adult SBP as the Outcome, and Adult Height and Adult BMI as Mediators eTable 1. Predictors of Systolic Blood Pressure in the Childhood Weight Model (Figure 3 in main text) eTable 2. Predictors of Height in the Childhood Weight Model (Figure 3 in Main Text) eTable 3. Predictors of Body Mass Index in the Childhood Weight Model (Figure 3 in Main Text) eTable 4. Predictors of Systolic Blood Pressure in the Childhood Height Model (Figure 4 in Main Text) eTable 5. Predictors of Height in the Childhood Height Model (Figure 4 in Main Text) eTable 6. Predictors of Body Mass Index in the Childhood Height Model (Figure 4 in Main Text) [file jamanetwopen-e2514289-s001.pdf]

# Supplemental Online Content

Strassmann BI, Vincenz C, Villamor E, Lovett JL, Dolo ZD, Shedden K. Risks and benefits of weight gain in children with undernutrition. *JAMA Netw Open*. 2025;8(6):e2514289. doi:10.1001/jamanetworkopen.2025.14289

**eMethods**

**eResults**

**eFigure 1.** Selected Goodness of Fit Plots for Multiple Imputation

**eFigure 2.** Principal Component (PC) Loadings for Weight and Height, Obtained Separately for Females and Males, Plotted as Functions of Age

**eFigure 3.** Minimally Adjusted Mediation Analysis Results with Childhood Weight as the Exposure, Adult SBP as the Outcome, and Adult Height and Adult BMI as Mediators

**eFigure 4.** Minimally Adjusted Mediation Analysis Results with Childhood Height as the Exposure, Adult SBP as the Outcome, and Adult Height and Adult BMI as Mediators

**eTable 1.** Predictors of Systolic Blood Pressure in the Childhood Weight Model (Figure 3 in main text)

**eTable 2.** Predictors of Height in the Childhood Weight Model (Figure 3 in main text)

**eTable 3.** Predictors of Body Mass Index in the Childhood Weight Model (Figure 3 in main text)

**eTable 4.** Predictors of Systolic Blood Pressure in the Childhood Height Model (Figure 4 in main text)

**eTable 5.** Predictors of Height in the Childhood Height Model (Figure 4 in main text)

**eTable 6.** Predictors of Body Mass Index in the Childhood Height Model (Figure 4 in main text)

This supplemental material has been provided by the authors to give readers additional information about their work.

© 2025 Strassmann BI et al. *JAMA Network Open*

**eMethods**

**Systolic Blood Pressure Measurement**

Sphygmomanometers were regularly calibrated against a mercury sphygmomanometer. Data were double entered and cleaned and the second and third measurements were averaged for use in the analyses. One author (CV) took all measurements in 2010 to April 2012, and another (ZDD), who shared the same ethnicity and native language as the participants, took all measurements from May 2012 to 2019. The SBP measurements were conducted indoors in private rooms in a non-clinical setting. We regressed the mean of the second and third SBP measurement for F0 mothers on maternal age and ambient temperature at measurement. The unstandardized residuals from this regression as well as from a similar regression for F0 fathers were adjusted for in the main models.

**Preparing anthropometric data**

Two persons from the same ethnicity as the participants, and who were fully trained, recorded the data manually on forms at the time of measurement; they then double-entered the data into Microsoft Excel. Other persons examined any discrepancies and corrected typographical errors by referring to the original field forms.

**Wealth Z-scores**

The wealth z-scores refer to the wealth of the participant's family at the time of their enrollment in the study. Wealth included land, livestock, income from labor or buying and selling, and rarely, remittances. These scores were relative to other families in the same village. For this purpose, a family was defined as a work-eat group (WEG), which means the persons who ate from the same millet harvest and who shared a head of household (Strassmann and Warner 1998). Household heads were usually male but could be an elderly woman who worked alone or accompanied by a grandchild. A WEG is the fundamental economic unit in Dogon villages. The steps involved in calculating relative WEG wealth in a village were as follows: 1. Made a list of the WEGs, identified by the name of their heads. 2. Trained raters (judges) in the protocols for evaluating relative wealth. A small village might have three raters and a large village, five raters. The average number of raters was four and there was always one rater who came from each patrilineage in a given village. The several raters worked simultaneously and independently with no opportunity for inter-rater communication. 3. Each rater independently rated each WEG 1 to 3 on wealth (1 = richest, 2 = middle, 3 = poorest), roughly balancing the size of each group into thirds. 4. Each rater then partitioned the WEGs in each group into the wealthiest and poorest half, turning the three groups into 6 groups. 5. Taking the six groups into consideration, the raters then ranked the WEGs in their village from 1 to N, with N referring to the total number of WEGs in the village. 6. The raters for a given village met together and arrived at a consensus ranking by going through steps 3 to 5 together and talking with each other. 7. The researchers transformed the data, such that instead of a uniform distribution from 1 to N for each village, the WEG ranks were standardized and followed a normal distribution with values ranging from approximately -2 standard deviations to +2 standard deviations. The wealth z-scores have been used in prior publications and their validity established by comparison to objective data (Strassmann 2011).

**Multiple Imputation**

To obtain body size measures at identical ages for analysis, childhood body measurements at integer ages 1 through 10 years were multiply imputed based on all directly observed measurements between ages 0 and 11 years. Multiple imputation was conducted using Gaussian-process regression based on the covariance models of Paciorek et al. (2006)[1], with parameters estimated using maximum likelihood. The modeling framework was extended to handle covariate effects on means, variances, and covariances via splines. We separately imputed anthropometry data (weight and height) for female and male subjects. Trajectories of correlated values at ages 1, 2, ..., 10 were imputed 20 times for each subject, using models fit to all observed data for ages up to 11. Means and variances were modeled with five degree of freedom splines, and correlations were modeled through a temporal smoothing parameter that was log-linear with age. To assess appropriateness of fit, plots of overlaid imputed and observed values were inspected (eFigure1 in Results).

© 2025 Strassmann BI et al. *JAMA Network Open*

**Regression Models**

The mean structure (fixed effects) for the mixed effects regression model with SBP as the dependent variable in terms of childhood weight is:

SBP = I(Village) + $age_x$ + $age_x^2$ + $age_x^3$ + log_num_meas + temp + wealth + cigarettes + preg_mo + $preg\_mo^2$ + breast_feeding_mo + mom_ht + mom_sbp + dad_ht + dad_sbp + height + weight + weight_pc + height*weight_pc + bmi*weight_pc

Notes: Village is categorical and I(.) indicates that it is converted to binary indicators; $age_x$ is (age - 10)/10 for numerical stability; log_num_meas is the base 10 logarithm of the number of times the subject has had a SBP measurement as of the current SBP measurement; wealth is a Z-scored wealth variable (see SI), cigarettes is the number of cigarettes smoked per week; preg_mo is the number of months elapsed in the current pregnancy, coded as zero if the individual is not currently pregnant; breast_feeding_mo is the number of months of continuous breastfeeding since the woman was last not breastfeeding, coded as zero if the individual is not currently breastfeeding; mom_ht and dad_ht are parental heights in centimeters; mom_sbp and dad_sbp are parental SBP values Z-scored relative to ambient temperature and age, stratified by sex; height and weight are the individual's current height and weight at the SBP measurement in cm; weight_pc is the principal component score for the individual's childhood weight trajectory.

The random effects structure for the SBP model is (1 | Id) + (0 + $age_x$ | Id) + (1 | mom_Id), which means that the model includes random intercepts for individuals (Id), random age slopes for individuals (on the transformed $age_x$ age scale), random intercepts for mothers, and residual variance.

The mediation analysis also involves fitting models for each of the mediators, height and BMI. The fixed effects mean structure for the BMI model is:

BMI = I(Village) + $age_x$ + $age_x^2$ + $age_x^3$ + log_num_meas + temp + wealth + cigarettes + preg_mo + $preg\_mo^2$ + breast_feeding_mo + mom_ht +dad_ht + height + weight + weight_pc

When predicting height, the same mean structure is used except that the pregnancy and breast-feeding terms are not included.

**eReferences**


1. Paciorek CJ, Schervish MJ. Spatial modelling using a new class of nonstationary covariance functions. *Environmetrics*. Aug 2006;17(5):483-506.
2. Strasssmann BI, Warner, J. Predictors of fecundability and conception waits among the Dogon of Mali. Am. J. Phys. Anthropol. 105:167-184.
3. Strassmann BI. 2011. Cooperation and competition in a cliff-dwelling people. *PNAS* 108:108984-10901.


© 2025 Strassmann BI et al. *JAMA Network Open*

**eResults**

**Prevalence of Pre-Hypertension and Hypertension**

We calculated the observed prevalence of pre-hypertension (SBP ≥ 120 mm Hg) and hypertension (SBP ≥ 140 mm Hg) in adults age ≥ 18 years upon last SBP measurement. The median (IQR) age was 21.14 (19.47, 23.14). The prevalence of pre-hypertension was 10.2% in females and 18.1% in males. The prevalence of hypertension was 1.0% in females and 1.7% in males.

## eFigure 1. Selected goodness of fit plots for multiple imputation.

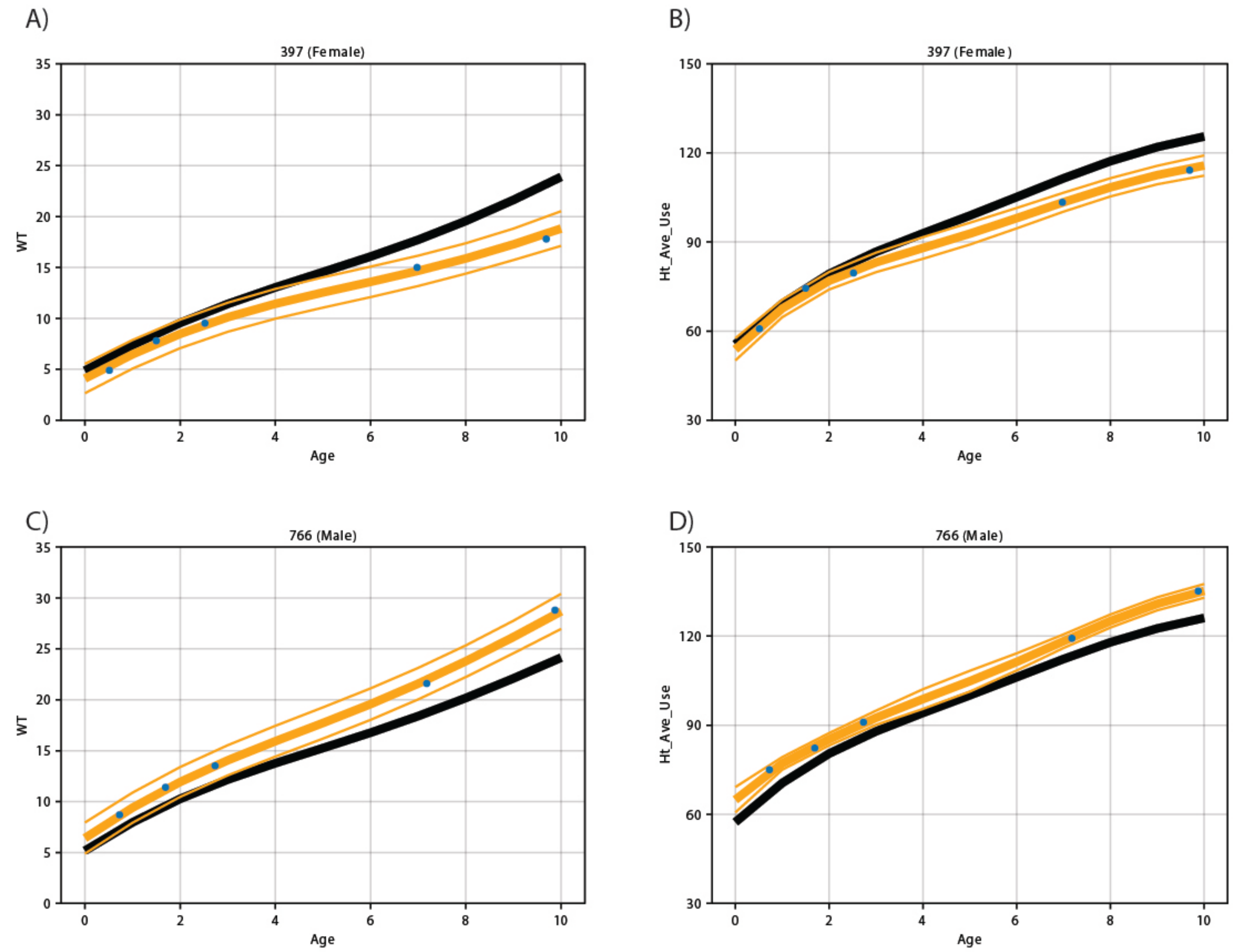


A. Weight (kg) for female ID = 397. B. Height (cm) for female ID = 397. Weight (kg) for male ID = 766. B. Height (cm) for male ID = 766. In each plot, the blue dots are the observed values, the heavy black curve is the sex-specific mean, the heavy orange curve is the mean of the imputation distribution for the given individual, and the two lighter orange curves are pointwise 95% ranges for the imputation distribution. We imputed 20 trajectories for each individual, and these imputed trajectories will primarily (with 95% probability) fall between the two light orange curves. The full set of 1348 plots (not shown) demonstrate that the imputation model is flexible enough to adapt to a range of observed growth patterns, such as consistently above the mean, on the mean, below the mean, catch-up, and catch-down patterns.

**eFigure 2. Principal component (PC) loadings for weight and height, obtained separately for females and males, plotted as functions of age.**

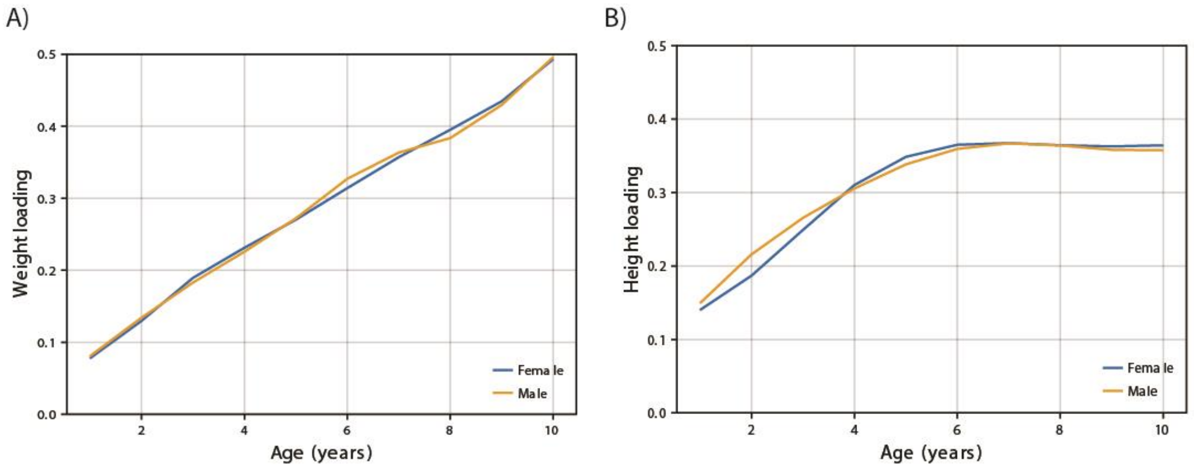


© 2025 Strassmann BI et al. *JAMA Network Open*

**eFigure 3. Minimally adjusted mediation analysis results with childhood weight as the exposure, adult SBP as the outcome, and adult height and adult BMI as mediators.**

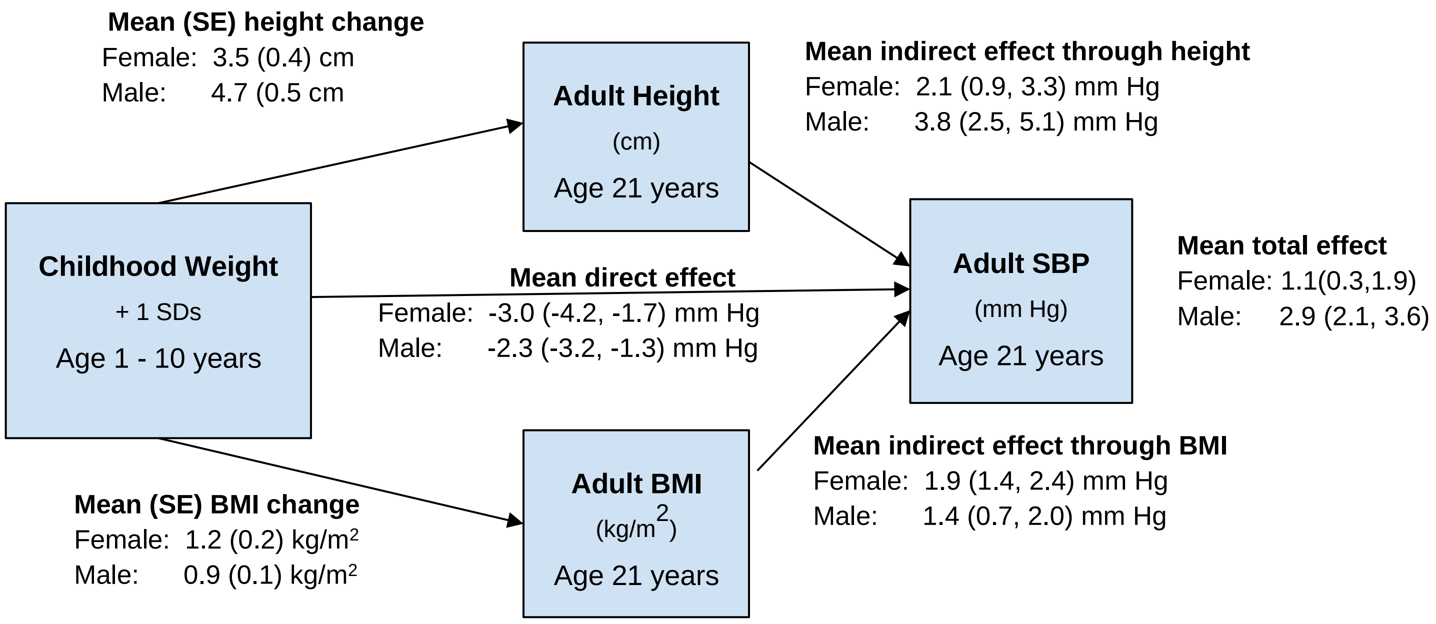


The exposure was modelled as being + 1 SD above the mean versus on the mean childhood weight trajectory, continuously from age 1 to 10 years. This exposure was age and sex specific, as well as internal to our data. The mediation outcome was adult SBP (mm Hg) at age 21 years and the mediators were adult height (cm) and adult BMI ($kg/m^2$) at age 21 years. This model was adjusted for age, village upon enrollment, log of the number of times SBP was taken, and temperature in degrees Celsius at SBP measurement.

© 2025 Strassmann BI et al. *JAMA Network Open*

**eFigure 4. Minimally adjusted mediation analysis results with childhood height as the exposure, adult SBP as the outcome, and adult height and adult BMI as mediators.**

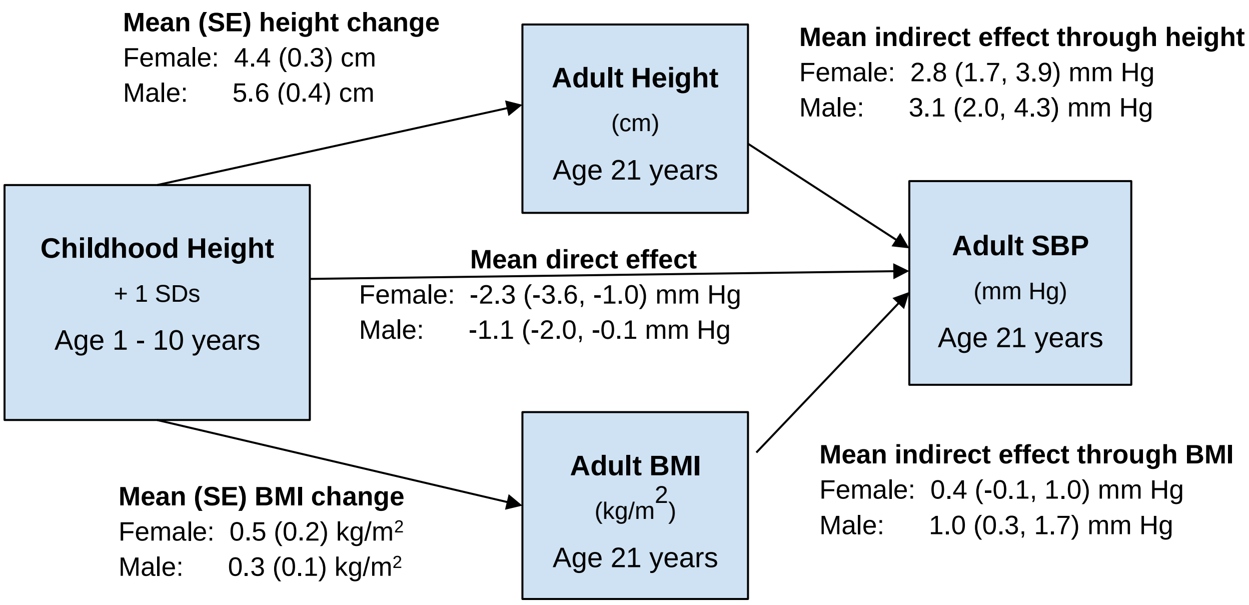


The exposure was modelled as being + 1 SD above the mean versus on the mean childhood height trajectory, continuously from age 1 to 10 years. This exposure was age and sex specific, as well as internal to our data. The mediation outcome was adult SBP (mm Hg) at age 21 years and the mediators were adult height (cm) and adult BMI (kg/$m^2$) at age 21 years. This model was adjusted for age, village upon enrollment, log of the number of times SBP was taken, and temperature in degrees Celsius at SBP measurement.

© 2025 Strassmann BI et al. *JAMA Network Open*

**eTable 1. Predictors of systolic blood pressure (SBP) in males (N = 501 participants, N = 3770 SBP measurements) and females (N = 433 participants, N = 3384 SBP measurements)(childhood weight model).**

| Childhood Weight Model | Males | | | Females | | |
|---|---|---|---|---|---|---|
| | ***β*** | ***SE*** | ***p*** | ***β*** | ***SE*** | ***p*** |
| Intercept | 147.571 | 15.224 | 0.0000 | 157.523 | 16.488 | 0.000 |
| Village 2 | 0.954 | 1.741 | 0.5838 | -1.006 | 1.532 | 0.5113 |
| Village 3 | -0.007 | 1.599 | 0.9967 | -1.328 | 1.720 | 0.4401 |
| Village 4 | 0.209 | 1.570 | 0.8939 | 0.173 | 1.631 | 0.9153 |
| Village 5 | -0.876 | 1.740 | 0.6146 | 0.090 | 1.656 | 0.9568 |
| Village 6 | -3.200 | 1.574 | 0.0421 | -4.830 | 1.468 | 0.0010 |
| Village 7 | -2.919 | 2.014 | 0.1474 | -2.804 | 1.908 | 0.1417 |
| Village 8 | -0.036 | 1.806 | 0.9842 | -0.624 | 1.676 | 0.7097 |
| Village 9 | -1.114 | 1.397 | 0.4250 | -4.285 | 1.332 | 0.0013 |
| Age_x[a] | -47.167 | 5.028 | 0.0000 | -48.473 | 7.229 | 0.0000 |
| Age_x squared [a] | 58.187 | 6.383 | 0.0000 | 44.815 | 7.873 | 0.0000 |
| Age_x cubed [a] | -20.183 | 2.798 | 0.0000 | -14.346 | 3.060 | 0.0000 |
| Log Number SBP Measurements | -15.824 | 0.941 | 0.0000 | -13.161 | 1.073 | 0.0000 |
| Temp °C | 0.015 | 0.031 | 0.6376 | 0.071 | 0.035 | 0.0453 |
| Wealth z-score | -0.680 | 0.406 | 0.0943 | -0.246 | 0.460 | 0.5918 |
| Number cigarettes per week [b] | -0.004 | 0.020 | 0.8290 | -- | -- | -- |
| Pregnancy (months) (centered) | -- | -- | -- | -1.592 | 0.324 | 0.0000 |
| Pregnancy (months) (centered) squared | -- | -- | -- | 0.170 | 0.047 | 0.0003 |
| Breastfeeding (months) (centered) | -- | -- | -- | -0.044 | 0.029 | 0.1311 |
| Mother's height (cm) | -0.079 | 0.069 | 0.2568 | -0.185 | 0.073 | 0.0117 |
| Mother's SBP z-score residuals | 0.076 | 0.024 | 0.0014 | 0.115 | 0.025 | 0.0000 |
| Father's height (cm) | -0.127 | 0.056 | 0.0242 | -0.046 | 0.062 | 0.4514 |
| Father's SBP z-score residuals | 0.010 | 0.019 | 0.6186 | 0.069 | 0.021 | 0.0009 |
| Height (cm) (centered) at SBP measurement | 0.458 | 0.037 | 0.0000 | 0.460 | 0.053 | 0.0000 |
| BMI (kg/m$^2$)(centered) at SBP measurement | 1.906 | 0.141 | 0.0000 | 1.906 | 0.104 | 0.0000 |
| Childhood Weight principal component score | -0.665 | 0.185 | 0.0003 | -0.991 | 0.231 | 0.0000 |
| Height centered at SBP x Weight principal component score | 0.023 | 0.008 | 0.0057 | 0.015 | 0.011 | 0.1582 |

© 2025 Strassmann BI et al. *JAMA Network Open*

| Childhood Weight Model | Males | | | Females | | |
|---|---|---|---|---|---|---|
| | ***β*** | ***SE*** | ***p*** | ***β*** | ***SE*** | ***p*** |
| BMI centered at SBP x childhood weight principal component score | -0.056 | 0.044 | 0.1945 | -0.020 | 0.027 | 0.4526 |
| Mother Variance | 0.235 | 0.099 | 0.0180 | 0.151 | 0.135 | 0.2657 |
| Participant Variance | 0.521 | 0.097 | 0.0000 | 0.680 | 0.142 | 0.0000 |
| Participant x Age_x[a] Variance | 0.011 | 0.002 | 0.0000 | 0.008 | 0.002 | 0.0000 |
| Residual variance | 45.753 | -- | -- | 48.777 | -- | -- |

[a]Age_x = (Age – 10) / 10 where Age is age in years

[b]Females did not smoke due to gender norms.

© 2025 Strassmann BI et al. *JAMA Network Open*

**eTable 2. Predictors of height (centered) in males and females at age 21 years (childhood weight model).**

| Childhood Weight Model | Males (N = 3773) | | | Females (N = 3387) | | |
|---|---|---|---|---|---|---|
| | ***β*** | ***SE*** | ***p*** | ***β*** | ***SE*** | ***p*** |
| Intercept | -88.033 | 4.570 | 0.0000 | -94.949 | 3.726 | 0.0000 |
| Village 2 | 1.109 | 0.528 | 0.0358 | -0.603 | 0.362 | 0.0960 |
| Village 3 | 1.178 | 0.483 | 0.0147 | -0.733 | 0.408 | 0.0722 |
| Village 4 | 1.042 | 0.485 | 0.0317 | -1.638 | 0.366 | 0.0000 |
| Village 5 | 1.342 | 0.538 | 0.0126 | -0.450 | 0.387 | 0.2451 |
| Village 6 | 2.071 | 0.488 | 0.0000 | -0.225 | 0.341 | 0.5089 |
| Village 7 | 1.147 | 0.621 | 0.0646 | 1.046 | 0.447 | 0.0193 |
| Village 8 | 0.946 | 0.535 | 0.0771 | -0.414 | 0.398 | 0.2984 |
| Village 9 | 0.890 | 0.432 | 0.0395 | 0.273 | 0.318 | 0.3901 |
| Age_x [a] | 48.052 | 3.200 | 0.0000 | 91.687 | 2.884 | 0.0000 |
| Age_x squared [a] | 15.618 | 4.503 | 0.0005 | -70.966 | 4.002 | 0.0000 |
| Age_x cubed [a] | -19.657 | 1.905 | 0.0000 | 17.226 | 1.675 | 0.0000 |
| Wealth z-score | 0.131 | 0.115 | 0.2549 | 0.631 | 0.107 | 0.0000 |
| Number cigarettes per week [b] | -0.004 | 0.013 | 0.7546 | -- | -- | -- |
| Mother’s height (cm) | 0.2372 | 0.021 | 0.0000 | 0.271 | 0.017 | 0.0000 |
| Father’s height (cm) | 0.212 | 0.017 | 0.0000 | 0.219 | 0.014 | 0.0000 |
| Childhood Weight principal component score | 1.304 | 0.048 | 0.0000 | 0.952 | 0.037 | 0.0000 |
| Residual variance | 31.129 | -- | -- | 22.028 | -- | -- |

[a]Age_x = (Age – 10) / 10 where Age is age in years
[b]Females did not smoke due to gender norms.

© 2025 Strassmann BI et al. *JAMA Network Open*

**eTable 3. Predictors of Body Mass Index (centered) in males and females at age 21 years (childhood weight model).**

| Childhood Weight Model | Males (N = 3773) | | | Females (N = 3387) | | |
|---|---|---|---|---|---|---|
| | ***β*** | ***SE*** | ***p*** | ***β*** | ***SE*** | ***p*** |
| Intercept | 10.531 | 1.173 | 0.0000 | 10.546 | 1.575 | 0.0000 |
| Village 2 | -0.401 | 0.134 | 0.0028 | 0.043 | 0.152 | 0.7766 |
| Village 3 | -0.582 | 0.124 | 0.0000 | -0.087 | 0.170 | 0.6117 |
| Village 4 | -0.132 | 0.125 | 0.2931 | 0.244 | 0.152 | 0.1081 |
| Village 5 | -0.215 | 0.138 | 0.1193 | -0.141 | 0.161 | 0.3812 |
| Village 6 | -0.644 | 0.125 | 0.0000 | 0.049 | 0.142 | 0.7316 |
| Village 7 | -0.634 | 0.158 | 0.0001 | 0.317 | 0.188 | 0.0907 |
| Village 8 | -0.698 | 0.137 | 0.0000 | -0.372 | 0.166 | 0.0252 |
| Village 9 | -0.472 | 0.110 | 0.0000 | 0.050 | 0.135 | 0.7119 |
| Age_x [a] | -1.506 | 0.825 | 0.0680 | 5.710 | 1.196 | 0.0000 |
| Age_x squared[a] | 12.868 | 1.161 | 0.0000 | 8.637 | 1.668 | 0.0000 |
| Age_x cubed [a] | -6.027 | 0.491 | 0.0000 | -5.641 | 0.698 | 0.0000 |
| Wealth z-score | 0.089 | 0.030 | 0.0026 | 0.1210 | 0.0448 | 0.0069 |
| Number cigarettes per week [b] | -0.004 | 0.003 | 0.2000 | -- | -- | -- |
| Pregnancy (months) (centered) | -- | -- | -- | 0.131 | 0.083 | 0.116 |
| Pregnancy (months) (centered) squared | -- | -- | -- | 0.012 | 0.012 | 0.3752 |
| Breastfeeding (months) (centered) | -- | -- | -- | -0.048 | 0.007 | 0.0000 |
| Mother's height (cm) | -0.025 | 0.006 | 0.0000 | -0.046 | 0.007 | 0.0000 |
| Father's height (cm) | -0.050 | 0.004 | 0.0000 | -0.047 | 0.006 | 0.0000 |
| Childhood Weight principal component score | 0.310 | 0.012 | 0.0000 | 0.462 | 0.015 | 0.0000 |
| Residual Variance | 2.074 | -- | -- | 3.700 | -- | -- |

[a]Age_x = (Age – 10) / 10 where Age is age in years
[b]Females did not smoke due to gender norms.

© 2025 Strassmann BI et al. *JAMA Network Open*

**eTable 4. Predictors of systolic blood pressure (SBP) in males (N = 501 participants, N = 3770 SBP measurements) and females (N = 433 participants, N = 3384 SBP measurements) (childhood height model).**

| Childhood Height Model | Males | | | Females | | |
|---|---|---|---|---|---|---|
| | ***β*** | ***SE*** | ***p*** | ***β*** | ***SE*** | ***p*** |
| Intercept | 151.0837 | 15.4529 | 0.0000 | 160.9507 | 16.7819 | 0.0000 |
| Village 2 | 0.9209 | 1.7505 | .5988 | -0.7634 | 1.5543 | 0.6233 |
| Village 3 | 0.1857 | 1.6084 | 0.9081 | -0.8048 | 1.7401 | 0.6437 |
| Village 4 | 0.3269 | 1.5789 | 0.8360 | 0.2069 | 1.6643 | 0.9011 |
| Village 5 | -0.7415 | 1.7528 | 0.6723 | 0.4097 | 1.6818 | 0.8075 |
| Village 6 | -3.0784 | 1.5818 | 0.0516 | -4.5767 | 1.4901 | 0.0021 |
| Village 7 | -2.7404 | 2.0260 | 0.1762 | -2.5516 | 1.9355 | 0.1874 |
| Village 8 | 0.0831 | 1.8171 | 0.9635 | -0.3530 | 1.7024 | 0.8357 |
| Village 9 | -1.1298 | 1.4042 | 0.4210 | -4.4132 | 1.3524 | 0.0011 |
| Age_x [a] | -45.7314 | 5.0712 | 0.0000 | -46.9781 | 7.4306 | 0.0000 |
| Age_x squared[a] | 58.7913 | 6.3441 | 0.0000 | 45.3336 | 8.0075 | 0.0000 |
| Age_x cubed [a] | -20.7619 | 2.7845 | 0.0000 | -14.9506 | 3.0821 | 0.0000 |
| Log No. SBP Measurements | -15.9235 | 0.9419 | 0.0000 | -13.1090 | 1.0796 | 0.0000 |
| Temp °C | 0.0150 | 0.0313 | 0.6328 | 0.0723 | 0.0353 | 0.0409 |
| Wealth z-score | -0.6990 | 0.4087 | 0.0872 | -0.1423 | 0.4669 | 0.7606 |
| Number cigarettes per week [b] | -0.0052 | 0.0201 | 0.7963 | -- | -- | -- |
| Pregnancy (months) (centered) | -- | -- | -- | -1.5754 | 0.3239 | 0.0000 |
| Pregnancy (months (centered) squared | -- | -- | -- | 0.1696 | 0.0470 | 0.0003 |
| Breastfeeding (months) (centered) | -- | -- | -- | -0.0532 | 0.0290 | 0.0668 |
| Mother’s height (cm) | -0.0955 | 0.0703 | 0.1744 | -0.1931 | 0.0746 | 0.0096 |
| Mother’s SBP z-score residuals | 0.0753 | 0.0238 | 0.0016 | 0.1205 | 0.0251 | 0.0000 |
| Father’s height (cm) | -0.1358 | 0.0569 | 0.0171 | -0.0649 | 0.0627 | 0.3004 |
| Father’s SBP z-score residuals | 0.0118 | 0.0195 | 0.5457 | 0.0733 | 0.0209 | 0.0005 |
| Height (cm) (centered) at SBP measurement | 0.4369 | 0.0396 | 0.0000 | 0.4469 | 0.0559 | 0.0000 |
| BMI (kg/m$^2$)(centered) at SBP measurement | 1.8423 | 0.1360 | 0.0000 | 1.7915 | 0.1006 | 0.0000 |
| Childhood Height principal component score | -0.3000 | 0.1815 | 0.0983 | -0.6932 | 0.2288 | 0.0024 |

© 2025 Strassmann BI et al. *JAMA Network Open*

| **Childhood Height Model** | **Males** | | | **Females** | | |
|---|---|---|---|---|---|---|
| | ***β*** | ***SE*** | ***p*** | ***β*** | ***SE*** | ***p*** |
| Height centered at SBP measurement x Childhood Height principal component score | 0.0138 | 0.0075 | 0.0661 | 0.0188 | 0.0101 | 0.0613 |
| BMI centered at SBP measurement x Childhood Height principal component score | 0.0102 | 0.0403 | 0.0143 | 0.0009 | 0.0268 | 0.9732 |
| Mother variance | 0.2479 | 0.1012 | 0.0143 | 0.1610 | 0.1381 | 0.2435 |
| Participant variance | 0.5194 | 0.0972 | 0.0000 | 0.7048 | 0.1437 | 0.0000 |
| Participant x age variance | 0.0104 | 0.0017 | 0.0000 | 0.0077 | 0.0017 | 0.0000 |
| Residual variance | 45.838 | -- | -- | 48.817 | -- | -- |

[a]Age_x = (Age – 10) / 10 where Age is age in years
[b]Females did not use stimulants due to gender norms.

© 2025 Strassmann BI et al. *JAMA Network Open*

**eTable5. Predictors of height (centered) in males and females at age 21 years (childhood height model).**

| Childhood Height Model | Males (N = 5185) | | | Females (N = 3387) | | |
|---|---|---|---|---|---|---|
| | *β* | *SE* | *p* | *β* | *SE* | *p* |
| Intercept | -63.436 | 4.168 | 0.0000 | -76.609 | 3.568 | 0.0000 |
| Village 2 | 0.250 | 0.500 | 0.6140 | -0.164 | 0.360 | 0.6474 |
| Village 3 | 0.585 | 0.473 | 0.2169 | -0.669 | 0.385 | 0.0826 |
| Village 4 | 0.965 | 0.489 | 0.0483 | -0.548 | 0.356 | 0.1236 |
| Village 5 | 0.562 | 0.500 | 0.0018 | -0.273 | 0.350 | 0.4354 |
| Village 6 | 1.150 | 0.469 | 0.0141 | -0.044 | 0.336 | 0.8963 |
| Village 7 | -0.296 | 0.567 | 0.6020 | 1.120 | 0.411 | 0.0065 |
| Village 8 | -0.149 | 0.526 | 0.7772 | 0.780 | 0.379 | 0.0395 |
| Village 9 | 0.498 | 0.417 | 0.2327 | 0.574 | 0.333 | 0.0854 |
| Age_x [a] | 48.519 | 2.922 | 0.0000 | 92.307 | 2.587 | 0.0000 |
| Age_x squared[a] | 15.410 | 4.117 | 0.0002 | -71.955 | 3.590 | 0.0000 |
| Age_x cubed [a] | -19.624 | 1.745 | 0.0000 | 17.740 | 1.503 | 0.0000 |
| Wealth z-score | 0.201 | 0.109 | 0.0643 | 0.440 | 0.097 | 0.0000 |
| Number cigarettes per week [b] | -0.005 | 0.012 | 0.6706 | -- | -- | -- |
| Mother's height (cm) | 0.158 | 0.020 | 0.0000 | 0.214 | 0.018 | 0.0000 |
| Father's height (cm) | 0.143 | 0.015 | 0.0000 | 0.163 | 0.013 | 0.0000 |
| Childhood Height principal component score | 1.615 | 0.039 | 0.0000 | 1.252 | 0.035 | 0.0000 |
| Residual Variance | 25.595 | -- | -- | 17.527 | -- | -- |

[a]Age_x = (Age – 10) / 10 where Age is age in years
[b]Females did not smoke due to gender norms.

© 2025 Strassmann BI et al. *JAMA Network Open*

**eTable6. Predictors of Body Mass Index (centered) in males and females at age 21 years (childhood height model).**

| Childhood Height Model | Males (N = 3773) | | | Females (N = 3387) | | |
|---|---|---|---|---|---|---|
| | *β* | *SE* | *p* | *β* | *SE* | *p* |
| Intercept | 6.9984 | 1.2453 | 0.0000 | 6.0557 | 1.7080 | 0.0004 |
| Village 2 | -0.4626 | 0.1390 | 0.0009 | -0.1079 | 0.1608 | 0.5019 |
| Village 3 | -0.7680 | 0.1303 | 0.0000 | -0.4762 | 0.1774 | 0.0073 |
| Village 4 | -0.2572 | 0.1296 | 0.0472 | 0.0937 | 0.1656 | 0.5715 |
| Village 5 | -0.2927 | 0.1411 | 0.0380 | -0.5277 | 0.1685 | 0.0017 |
| Village 6 | -0.8012 | 0.1307 | 0.0000 | -0.2263 | 0.1539 | 0.1415 |
| Village 7 | -0.8359 | 0.1640 | 0.0000 | 0.1642 | 0.1984 | 0.4077 |
| Village 8 | -0.8489 | 0.1476 | 0.0000 | -0.6874 | 0.1765 | 0.0001 |
| Village 9 | -0.4811 | 0.1151 | 0.0000 | 0.0917 | 0.1394 | 0.5107 |
| Age_x [a] | -1.8805 | 0.9136 | 0.0396 | 6.2254 | 1.3445 | 0.0000 |
| Age_x squared[a] | 13.1044 | 1.2887 | 0.0000 | 7.6035 | 1.8807 | 0.0001 |
| Age_x cubed [a] | -6.0542 | 0.5455 | 0.0000 | -5.1099 | 0.7871 | 0.0000 |
| Wealth z-score | 0.1100 | 0.0319 | 0.0006 | 0.0565 | 0.0476 | 0.2353 |
| Number Cigarettes per week [b] | -0.0025 | 0.0037 | 0.4982 | -- | -- | -- |
| Pregnancy (mo) (centered) | -- | -- | -- | 0.1009 | 0.0944 | 0.2849 |
| Pregnancy (mo) (centered)**2 | -- | -- | -- | 0.0171 | 0.0137 | 0.2120 |
| Breastfeeding (months) (centered) | -- | -- | -- | -0.0380 | 0.0077 | 0.0000 |
| Mother’s height (cm) | -0.0093 | 0.0057 | 0.0998 | -0.0354 | 0.0076 | 0.0000 |
| Father’s height (cm) | -0.0430 | 0.0045 | 0.0000 | -0.0301 | 0.0063 | 0.0000 |
| Childhood Height principal component score | 0.1422 | 0.0123 | 0.0000 | 0.2155 | 0.0172 | 0.0000 |
| Residual variance | 2.572 | -- | -- | 4.817 | -- | -- |

[a]Age_x = (Age – 10) / 10 where Age is age in years
[b]Females did not smoke due to gender norms.

© 2025 Strassmann BI et al. *JAMA Network Open*
