# Supplementary material for: Risks and Benefits of Weight Gain in Children With Undernutrition
Source: JAMA Netw Open. 2025 Jun 6;8(6):e2514289. doi: 10.1001/jamanetworkopen.2025.14289 (PMC12144626; doi:10.1001/jamanetworkopen.2025.14289)
Supplement: Supplement 2. — Data Sharing Statement [file jamanetwopen-e2514289-s002.pdf]

Data Sharing Statement

Strassmann. Risks and Benefits of Weight Gain in Childhood Undernourishment. *JAMA Netw Open*. Published June 06, 2025. doi:10.1001/jamanetworkopen.2025.14289

Data
Data available: No

Additional Information
Explanation for why data not available: The data are not currently publicly available for distribution, but a future public data set will be made available. Researchers interested in the data are asked to contact the corresponding author with a proposal.
